# Exposure of Domestic Cats (*Felis catus*) to Rodenticidal Compounds

**DOI:** 10.3390/toxics13080663

**Published:** 2025-08-07

**Authors:** Vesna Cerkvenik-Flajs, Detlef Schenke, Simona Korenjak-Černe, Anton Perpar, Jens Jacob, Susanne Schwonbeck, Sven Kleine Bardenhorst, Torsten Hahn, Marko Cvetko, Mitja Gombač

**Affiliations:** 1 Veterinary Faculty, Institute of Pathology, Wild Animals, Fish and Bees, University of Ljubljana, Gerbičeva 60, SI-1000 Ljubljana, Slovenia; marko.cvetko@vf.uni-lj.si (M.C.); mitja.gombac@vf.uni-lj.si (M.G.); 2 Julius Kühn Institute (JKI)—Federal Research Centre for Cultivated Plants, Institute for Ecological Chemistry, Plant Analysis and Stored Product Protection, Königin-Luise Str. 19, D-14195 Berlin, Germany; detlef.schenke@julius-kuehn.de; 3 School of Economics and Business, University of Ljubljana, Kardeljeva ploščad 17, SI-1000 Ljubljana, Slovenia; simona.cerne@ef.uni-lj.si; 4 Institute of Mathematics, Physics and Mechanics, Jadranska ulica 19, SI-1000 Ljubljana, Slovenia; 5 Biotechnical Faculty, Department of Agronomy, University of Ljubljana, Jamnikarjeva 101, SI-1000 Ljubljana, Slovenia; anton.perpar@bf.uni-lj.si; 6 Julius Kühn Institute (JKI)—Federal Research Centre for Cultivated Plants, Institute for Epidemiology and Pathogen Diagnostics, Rodent Research, Toppheideweg 88, D-48161 Münster, Germany; jens.jacob@julius-kuehn.de; 7 Fraunhofer Institute for Toxicology and Experimental Medicine ITEM, Nikolai-Fuchs-Str. 1, D-30625 Hannover, Germany; susanne.schwonbeck@item.fraunhofer.de (S.S.); torsten.hahn@item.fraunhofer.de (T.H.); 8 Institute of Epidemiology and Social Medicine, University of Münster, Albert-Schweitzer-Campus-1, D-48149 Münster, Germany; kleineba@uni-muenster.de

**Keywords:** anticoagulant toxicants, α-chloralose, domestic cats, environmental monitoring, mass spectrometry, risk assessment

## Abstract

Anticoagulant rodenticides (ARs) are highly effective, but can be of environmental concern due to primary and secondary non-target exposure, with the latter possible being relevant to domestic cats. Therefore, liver residues of ARs and an alternative rodenticide, α-chloralose, were systematically monitored in domestic cats for the first time in the current study. In 2021 and 2022, the carcasses of 99 cats were collected in Slovenia and liver residues were measured by using solid supported liquid–liquid extraction and LC-MS/MS. The results show that 65% of cats carried at least one rodenticide. The second-generation ARs brodifacoum and bromadiolone were most prevalent and found in 53.5 and 25.3% of the samples, respectively. Of first-generation ARs, coumatetralyl was the most prevalent (21.2% of cats). More compounds were detected at high human population density, low farm density and in rural versus intermediate landscapes, but no effect was found for livestock density. Similar trends were found for the presence of brodifacoum, bromadiolone and all rodenticides combined. Farm density was negatively correlated with brodifacoum liver concentration. Individual factors (cat age, sex, outdoor activity) did not matter. The results indicate that a reasonably populated rural landscape, and not the rural or intermediate environment as such, is the main driver of cat exposure to ARs. The risk quotient (RQ) of worst-case acute brodifacoum poisoning was 1506. In summary, a potential environmental problem is globally highlighted for cats that is probably related to secondary exposure to ARs, with a pattern different to that seen in wild predators. Cats are an appropriate sentinel species for assessing rodenticide exposure and endangerment in the environment.

## 1. Introduction

Anticoagulant rodenticides (ARs) are commonly used to control rodent populations in urban, suburban and agricultural areas. Although they are not authorised as pesticides in the European Union (EU), ARs are used as biocidal products and are regulated by the Biocidal Products Regulation (EU) 528/2012 [1]. ARs are vitamin K antagonists that inhibit the activity of the enzyme vitamin K epoxide reductase complex (VKORC) subunit 1, which converts vitamin K 2,3-epoxide into a biologically active form of vitamin K that is important for effective blood coagulation [2]. ARs are highly effective, easy to use, and prevent bait shyness; moreover, an antidote (vitamin K1) is available [3]. They are typically used in urban and peri-urban areas, often to control commensal rodents such as house mice (*Mus musculus*), Norway rats (*Rattus norvegicus*) and house rats (*Rattus rattus*).

Depending on their structure and function, ARs are divided into first and second generations—FGARs and SGARs, respectively. The latter, also known as superwarfarins, were introduced in response to the emerging resistance of pest rodents to FGARs. Due to the extended side chain at the 13th carbon atom of the hydroxycoumarin molecule with substituted phenyl rings and the increased hydrophobicity, SGARs have significantly improved activity and toxicity compared to FGARs. They are more effective (up to 100-fold more potent than warfarin), act over a significantly longer period of time and have a greater bioaccumulation potential, i.e., they remain in organisms and the environment for longer [4]. Repeated exposure to ARs can more easily result in a lethal dose due to the long half-life [5], but a single dose of an SGAR is also effective.

Several mitigation measures have been put in place by the authorities in many countries to minimise primary and secondary exposure of non-target individuals to ARs, and SGARs in particular [6]. These include the restriction of use to in and around buildings, application in tamper-resistant bait boxes, use by trained professionals only, and rapid removal of dead rodents. However, it is not always possible to apply these measures in low- and middle-income countries [7].

The numerous reports of non-target wildlife species, particularly birds and mammals, being endangered by primary and secondary exposure to ARs indicate an environmental risk to these animals, mainly in the form of sub-toxic/chronic or sublethal exposure [8]. Primary exposure is relevant in animals small enough to enter the bait stations, such as non-target rodents [9,10], passerine birds [11] and invertebrates [12]. Secondary exposure through consumption of rodenticide-loaden rodents may be widespread, reaching high-level felid carnivores; in a study carried out in urban southern California, ARs were found in 90% of bobcats (*Lynx rufus*) and mountain lions (*Puma concolor*). It has also been hypothesised that contamination with ARs causes mountain lions to become more susceptible to ectoparasitic infection with notoedric mange [13].

The effects of ARs on both target and non-target organisms are complex beyond mortality. A study of genome-wide expression in bobcats permanently exposed to SGARs and sublethally intoxicated revealed the altered expression of genes affecting not only haemostasis, but also xenobiotic metabolism, endoplasmic reticulum stress response, epithelial integrity, wound healing, and adaptive and innate immunity [14].

The exposure of pets to ARs is often unintentional, and in domestic cats (*Felis catus*), primary exposure to rodenticides usually occurs only in cases of clinical poisoning [15]. For example, of the calls received by the French National Poisons Information Centre, approximately 11% are related to AR exposure or poisoning, of which 9.5% involve domestic cats [5]. Cats may be more prone to secondary AR exposure than dogs [16] because of their feeding habits when roaming outdoors. Deliberate secondary poisoning has even been used in the past as a control strategy against feral cats and mustelid pests in New Zealand, mainly using rodents poisoned with brodifacoum [17,18,19]. The livers of cats found dead during these campaigns contained brodifacoum residues ranging from 1400 to 3730 ng/g.

Studies of the asymptomatic exposure of companion animals to rodenticides are scarce and mainly relate to dogs and cats, for which exposure has generally been observed to be lower than in wild animals [20,21,22,23]. α-Chloralose, although not an AR, was also considered as there are increasing reports of poisoning in dogs and cats with this substance [24,25,26,27]. The LD_50_ dose of α-chloralose is 100 mg/kg in cats, but 600–1000 mg/kg in dogs [28].

The number of cats in Slovenia has not yet been precisely determined, but EU statistics estimated it to be around 455,000 in 2022 [29]. This estimate is generally in line with the number of households, of which it is estimated that around 20% have at least two cats [30,31]. In practice, estimating the number of cats is difficult, because there are cats that live exclusively indoors with their owners, and others that roam unattended in the neighbourhood, i.e., “barn cats” that live uncontrolled and give birth to feral offspring. Cat colonies often develop in urban centres and suburban settlements, which may or may not be fed by humans. The roaming cat population is therefore divided into owned, semi-owned and unowned sub-populations [32].

Cats are opportunistic feeders, so predation on wildlife, especially small mammals and birds, is important for stray cats. Small mammals such as voles, mice and shrews (68%), followed by small birds such as passerines (21%) and reptiles such as lizards (8%), are most commonly delivered to the homes of their owners [33]. However, the relative proportions of mice and voles and the relative proportions of shrews and rodents vary among domestic cats in rural areas of central Poland [34]. Regular access to food provided by humans does not suppress the hunting behaviour of cats; instead, the time cats spend outdoors is an important determinant of predation [35].

The aim of the present study was to evaluate the effects of various landscape factors in Slovenia (human population density, density of farms, rural versus intermediate habitat, livestock density) as well as the individual parameters of cats (age, sex, outdoor versus indoor–outdoor activity) on the presence and concentration of eight AR compounds and α-chloralose in cat liver samples. In wild terrestrial vertebrates such as foxes [36,37], stone martens (*Martes foina*), pine martens (*Mustela putorius*) [38] and European hedgehogs (*Erinaceus europaeus*) [39], brodifacoum and bromadiolone are highly prominent in liver samples. We hypothesised that this would also be the case in cats due to secondary exposure. Furthermore, we hypothesised that (1) outdoor activity but not the age and sex of cats would be drivers of rodenticide residue presence and concentration. Moreover, based on findings in several species [23] we expected that (2) livestock density would play a role; and based on studies of other terrestrial and avian predators [36,37,38,39], we hypothesised that (3) an intermediate habitat which includes an urban area would lead to a more frequent presence of rodenticide residues, and thus to a higher residue concentration in cats.

## 2. Materials and Methods

### 2.1. Sample Collection

**Figure 1:**
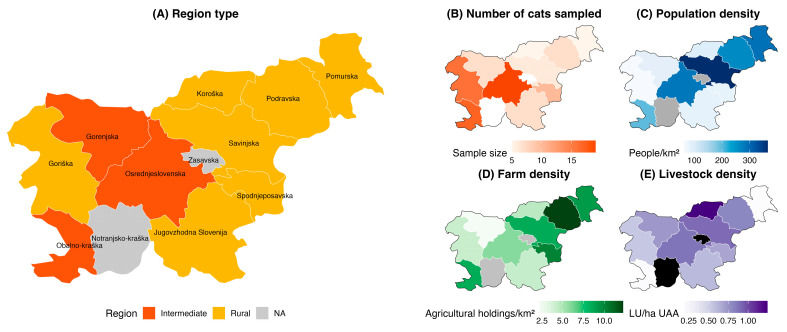
Statistical regions of Slovenia according to (**A**) region type, based on degree of urbanisation (intermed-intermediate type, rural–predominantly rural type), (**B**) number of cats sampled, (**C**) population density, (**D**) farm density and (**E**) livestock density (LU—livestock unit, UAA—utilised agricultural area) [42,43,44].

In 2021 and 2022, the carcasses of 99 domestic cats were obtained from veterinary clinics, animal shelters and the Veterinary Hygiene Service (i.e., roadkill) from ten geographical regions of Slovenia: Mura–Pomurska (*n* = 5), Drava–Podravska (*n* = 7), Savinja–Savinjska (*n* = 6), Lower Sava–Spodnjeposavska (*n* = 10), Southeast Slovenia–Jugovzhodna Slovenija (*n* = 7), Carinthia–Koroška (*n* = 5), Central Slovenia–Osrednjeslovenska (*n* = 19), Upper Carniola–Gorenjska (*n* = 7), Gorizia–Goriška (*n* = 16) and Coastal Karst–Obalno-kraška (*n* = 17). The statistical regions of Slovenia in connection with the environmental characteristics and the number of sampled cats are shown in Figure 1 [40,41,42,43,44].

The cat carcasses were frozen after death or after they were found dead and transported to the Institute of Pathology, Wild Animals, Fish and Bees of the Veterinary Faculty of the University of Ljubljana, Slovenia, where they were necropsied immediately after thawing. During necropsy, liver samples were collected and stored for 0.5–15 months at −20 °C and for three weeks at −80 °C to avoid contamination with viable *Echinococcus* eggs. The samples were transported in dry ice to the Julius Kühn Institute in Berlin, Germany, where they were stored at −80 °C until chemical analysis. All samples were collected post-mortem, and thus approval from the national ethics committee/social welfare agency was not required.

The sex, age category, body weight, cause of death, ranging behaviour and location of each animal were recorded via the questionnaire filled out by the owners designed by vets (Table S1). If the cats predominantly lived indoors but had free outdoor access, they were categorised as indoor–outdoor animals. Of the 99 cats included in the study, 56 were male, and 43 were female. The cats were divided into five age categories: ≤1 year (*n* = 6), 1–5 years (*n* = 40), 6–10 years (*n* = 27), 11–15 years (*n* = 17) and >15 years (*n* = 7) (no data for *n* = 2). Body mass ranged from 0.75 to 9.7 kg, with an average of 3.36 kg. Most (*n* = 61) were outdoor cats and 26 were indoor–outdoor cats (no data were available for 12 cats, but these were not purely indoor cats as outdoor activity was a prerequisite for inclusion in the study).

### 2.2. Chemical Residue Analysis

For the residue analysis of eight ARs and α-chloralose, sample preparation was carried out as described in Cerkvenik-Flajs et al. [37]. In brief, a portion of the thawed cat liver sample was weighed and fortified with a surrogate mixture consisting of 100 ng each of acenocoumarol and diphacinone-d4. The homogenate from the chopping process was then put in methanol/water (2:1, *v*/*v*) and centrifuged. An aliquot of the supernatant was purified by using a Chem Elut diatomaceous earth (Agilent Technologies, Santa Clara, CA, USA) solid-support liquid extraction cartridge (unbuffered, 20 mL). A portion of the eluate was carefully evaporated and the dry residue was redissolved with a methanol/water mixture (1:1, *v*/*v*) containing the internal standards chlorophacinone-d4 and warfarin-d5. The subsequently filtered solution was stored at −20 °C until measurement.

Measurements of ARs were performed via liquid chromatography–electrospray tandem mass spectrometry (LC-ESI-MS/MS), using a 1290 Infinity II liquid chromatograph (Agilent Technologies, Santa Clara, CA, USA) coupled to the QTRAP 6500+ high performance triple quadrupole/linear ion trap mass selective detector (SCIEX, Framingham, MA, USA). This method is also suitable for the analysis of α-chloralose. Identification and quantification of the rodenticides were carried out with two characteristic precursor (Q1)–product ion (Q3) transitions of each rodenticide (except chlorophacinone) via Multiple Reaction Monitoring (Table S2) and confirmed by the acquisition of Enhanced Product Ion spectra in the ion trap mode of the mass spectrometer (Table S3).

Recovery samples were included in each batch for quality control. Wild boar liver was used as the matrix and fortified with 100 ng/g of each analyte (Table S4). No interferences below the reporting limit (RL) were detected in the blank quality control samples. All liver samples were fortified with the surrogate mixture for ongoing validation of the analytical performance (Table S5). All samples were measured twice, and the mean values were used for further analyses. The calibration curves were linear with r^2^ > 0.99 over the entire concentration range of matrix-matched standards (0.01–20.0 ng/mL). The RLs refer to the lowest calibration level with a signal-to-noise ratio of >6:1 and relative standard deviation of <20% in the sequence. The concentrations of the rodenticides were neither surrogate nor recovery-corrected.

### 2.3. Data Preparation and Methods for Statistical Analysis

The individual concentrations of rodenticides in the livers of the cats are presented in Table S1. The presence and concentration of each rodenticide and the pooled values across all rodenticides were evaluated using descriptive statistics (frequencies, percentages, minimum, maximum, median, mean, standard deviation to measure variability between individual samples). Based on Geduhn et al. [36], five concentration categories were used (I: not detected (n. d.); II: n. d. < c < 200 ng/g; III: 200 ng/g ≤ c < 800 ng/g; IV: 800 ng/g ≤ c < 2000 ng/g; V: c ≥ 2000 ng/g).

Since the collection of information on the related municipality allowed us to examine possible relationships with other external variables, we have supplemented the data with information on statistical regions, cohesion regions (eastern and western Slovenia) and groups of statistical regions based on the degree of urbanisation. At the landscape level, human population density per km^2^, the number of farms per km^2^ of municipal area, region type according to the international typology of the degree of urbanisation (intermediate versus rural) and the number of livestock units per ha of utilised agricultural area were used [40,42,43,44]. Except for the latter, parameters were grouped in three classes based on the respective 33rd and 66th percentiles as cut-off points. At the individual level, age (five age classes, see above), sex and activity (outdoor versus indoor–outdoor) were used.

The effects of the landscape and individual parameters on the number of rodenticides in liver tissue and the presence of rodenticides (for brodifacoum, bromadiolone, all rodenticides combined) were tested using Poisson or logistic regression models, respectively. The effects of landscape (for brodifacoum alone, as there were insufficient data for bromadiolone) and individual animal parameters (age, sex and activity for brodifacoum, but only age and sex for bromadiolone, as there were insufficient data for activity) on the concentration of rodenticide residues in liver tissue were tested using linear regression with log-transformed concentration measures. To ensure robust estimation, effect estimates and confidence intervals were derived from bootstrapped samples (b = 10,000) [45].

Statistical analysis was performed in R version 4.2.3 [46].

### 2.4. Risk Assessment for Acute Toxicity Using the Example of Brodifacoum

The authorisation of biocidal products, as regulated by Regulation (EU) 528/2012 [1], also includes an environmental risk assessment (ERA) of the potential risks posed by such products. The requirements for the ERA, including the assessment of risks due to secondary poisoning by ARs, are laid out in several technical guidelines [47,48]. The potential risks of the active substances in the biocidal products are evaluated using a tiered approach, depending on the amount and quality of data available. The predicted environmental concentration (PEC) is calculated based on the intended use of the biocidal product. The PEC is compared to the threshold of effects (predicted no-effect concentration, PNEC) on non-target organisms, in our case cats, which results in a risk quotient (RQ). If the RQ is below 1, the biocidal product is considered to pose no unacceptable risk to the environment.

In the present case of secondary poisoning in cats, we used acute toxicity data from the literature (lowest LD_50_ = 0.25 mg/kg) [49] and compared these to the predicted environmental concentrations (PECs) in mg/kg body weight (bw) as calculated according to the Emission Scenario Document (ESD) [48,50]. The calculations are provided in detail in the Supplementary Materials (Tables S8–S11).

## 3. Results

### 3.1. Necropsy

During necropsy, the cause of death or the reason for euthanasia was determined for 87 cats. These cats died or were euthanised due to blunt force trauma, mainly from motor vehicles (*n* = 21) and chronic kidney or liver failure (*n* = 19), followed by chronic heart failure (*n* = 9), neoplasms and feline panleukopenia (*n* = 6 each), feline leukaemia virus (FeLV) (*n* = 5), pyothorax and pneumonia (*n* = 3), feline infectious peritonitis (FIP), feline immunodeficiency virus (FIV) and collapse during ovariohysterectomy (*n* = 2 each), or gastric constipation, cystitis and purulent ophthalmitis (*n* = 1 each). Seven cats were euthanised due to old age and severe cachexia, while in three cats the presumed cause of death was poisoning with an unknown toxic substance: (1) (Table S1, sample ID 3) intoxication with ARs was suspected due to unclotted blood in the thoracic cavity, but the measured AR concentrations in the liver refuted this assumption, as the summed AR was 25.7 ng/g; (2) (Table S1, sample ID 41) the cause of death was trauma due to a ruptured pericardium; and (3) (Table S1, sample ID 59) intoxication with the molluscicide metaldehyde was confirmed.

### 3.2. Rodenticide Residues

**Figure 2:**
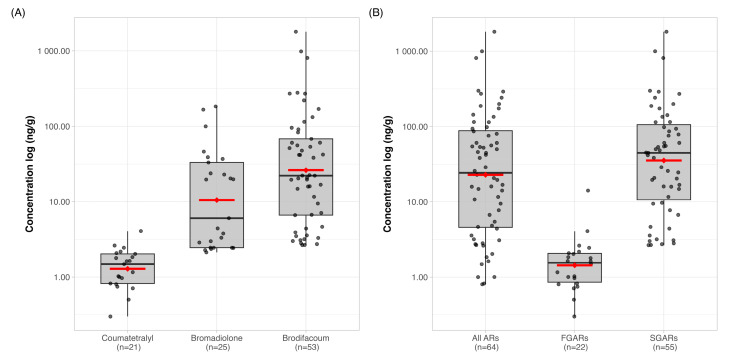
Distribution of rodenticide concentrations in cat liver samples on log scale. Red lines indicate mean concentrations and dots indicate values of individuals. (**A**) Concentrations of individual anticoagulant rodenticides (ARs) detected most frequently. (**B**) Summed concentrations by rodenticide generation (FGARs = first-generation ARs, SGARs = second-generation ARs). All ARs = sum of all ARs for those cats that contained at least one AR. FGARs = sum of all first-generation ARs (2 FGARs) for those cats that contained at least one FGAR. SGARs = sum of all second-generation ARs (4 SGARs) for those cats that contained at least one SGAR.

Residues of at least one tested rodenticide substance were detected in 64.6% of cats. The concentrations of the pooled ARs ranged from 0.8 to 1819.7 ng/g with a mean and median value of 110.8 and 24.3 ng/g, respectively. Just under half of the samples (48.4%) with AR residues contained one AR, 34.4% contained two ARs, 14.1% contained three ARs, while residues of four ARs were detected in 2.0% (Figure 2, Table S6).

The SGARs brodifacoum, bromadiolone, difenacoum and difethialone were found in 53.5, 25.3, 8.1 and 2.0% of the samples, respectively. We determined brodifacoum at concentrations of ≥800 ng/g in 3 of the 99 cat livers (3.0%), and 1 of these (1.0%) contained residues of >1800 ng/g. These three samples were found in the predominantly rural regions, from where the only sample with α-chloralose also originated. Among FGARs, coumatetralyl was the one most commonly found in the samples, at 21.2%, of which all cases were ≤4.1 ng/g. Warfarin and α-chloralose were each found in one sample (1.0%) with a concentration of 14.2 and 561.7 ng/g, respectively (Figure 2, Table S6).

**Figure 3:**
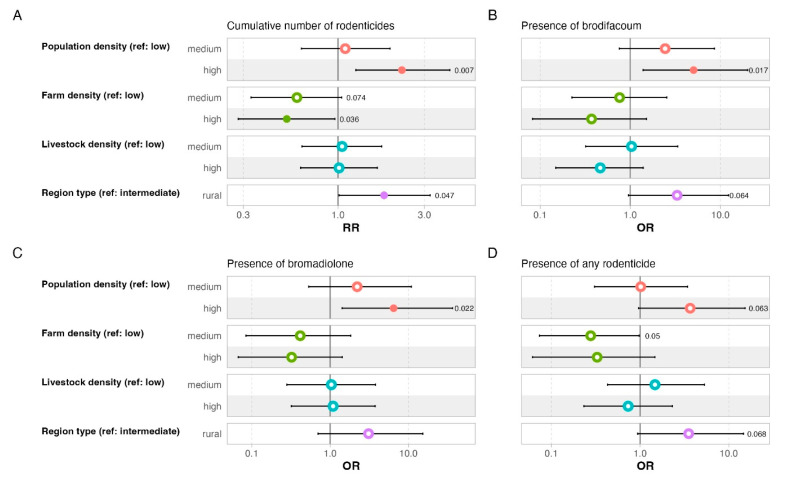
Effects of landscape variables on (**A**) number of rodenticidal compounds and presence of residues of (**B**) brodifacoum, (**C**) bromadiolone and (**D**) all rodenticides combined in liver samples of cats (*n* = 99). Colors refer to variables listed on the left. Filled dots indicate statistically significant effects (*p* < 0.05). *p*-values are presented only for *p* < 0.1. RR—rate ratio; OR—odds ratio.

More anticoagulant compounds were present in cats at locations with high versus low human population density (rate ratio [RR] = 2.25, *p* = 0.007), with the results indicating a more than twofold increase. In contrast, high farm density was associated with a 48% reduction in the number of compounds detected (RR = 0.52, *p* = 0.036), and the results were marginally statistically significant at medium versus low farm density (RR = 0.59, *p* = 0.075). An 80% increase in rodenticidal compounds was found for cats at locations with rural versus intermediate region type (RR = 1.8, *p* = 0.047) (Figure 3), but there was no effect of livestock density. This general pattern was similar for the presence of brodifacoum, bromadiolone and all rodenticides combined. Samples from areas with high population density were about five times more likely to be brodifacoum-positive compared to those from low-population-density areas (Odds Ratio [OR] = 5, *p* = 0.017), and a similar, statistically marginal positive trend was observed for rural habitats compared to those with intermediate region type (OR = 3.3, *p* = 0.064). Likewise, more bromadiolone-positive samples were present in cats at locations with high versus low human population density (OR = 6.5, *p* = 0.022), and there was a trend for an increased presence of all rodenticides combined at sites with high versus low human population density (OR = 3.7, *p* = 0.063) as well as those with rural versus intermediate region type (OR = 3.5, *p* = 0.068).

The only landscape parameter that mattered for brodifacoum concentration was farm density, with brodifacoum concentration lower at high versus low farm density (*p* = 0.03). In addition, there was a statistical trend of a higher brodifacoum concentration for cats found in rural versus intermediate habitats (*p* = 0.07) (Table 1).

**Figure 4:**
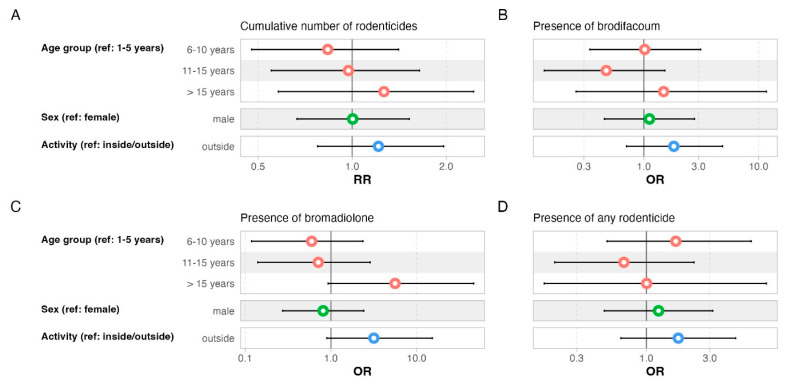
Effects of individual-level variables on (**A**) number of rodenticidal compounds and presence of residues of (**B**) brodifacoum, (**C**) bromadiolone and (**D**) all rodenticides combined in liver samples of cats (*n* = 86). Colors refer to variables listed on the left. Filled dots indicate significant effects (*p* < 0.05). RR—rate ratio; OR—odds ratio.

None of the individual parameters mattered for the number of rodenticides in liver tissue or the presence of brodifacoum, bromadiolone or all rodenticides combined (Figure 4). There was no effect of individual parameters on the concentrations of brodifacoum and bromadiolone (Table 2 and Table 3).

**Figure 5:**
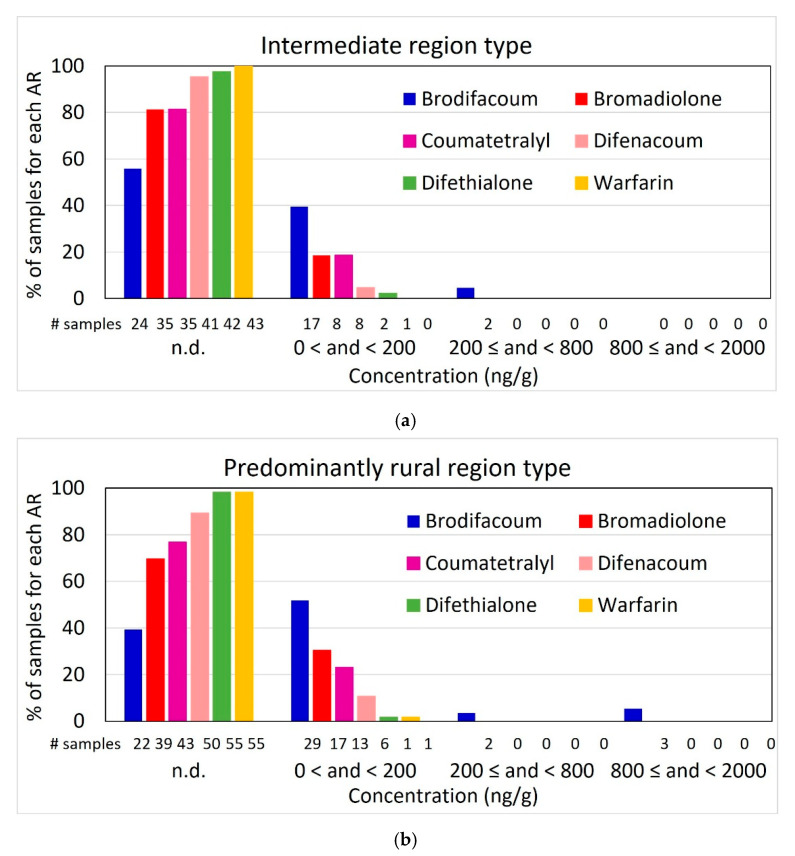
Residues of ARs in 99 domestic cat liver samples in Slovenia in 2021 and 2022 by region type ((**a**) intermediate, *n* = 43; (**b**) predominantly rural, *n* = 56) with percentage and number of samples for each AR per concentration category; #—number, n.d.—not detected.

Of the Slovenian regions for which data were collected, only three are classified as intermediate (Gorenjska, Obalno-kraška and Osrednjeslovenska), while all others (Goriška, Jugovzhodna Slovenija, Koroška, Podravska, Pomurska, Savinjska and Spodnjeposavska) are predominantly rural [44]. In the intermediate regions, 22 out of 43 samples were AR-positive (51.2%), while in the predominantly rural regions, 42 out of 56 samples were AR-positive (75.0%). The outdoor male with α-chloralose was found in the predominantly rural region of Goriška (Figure 5, Table S7).

### 3.3. The Results of the Risk Assessment for Acute Toxicity Using the Example of Brodifacoum

It is evident that the risk of being poisoned by primary and secondary poisoning is very high for non-target organisms, with the RQs for brodifacoum ranging from 15,000 to 855,855 [51]. However, specific toxicity data for cats are very limited. Following the procedure as detailed in the Emission Scenario Document (ESD), it is assumed that a predator prey (i.e., a target animal mouse or rat) forages on bait for only five consecutive days and is then caught by the predator (in the present case, a cat) immediately after their last meal on day five [48]. It is further assumed that rodents consume 10% of their body weight in food per day, and that a cat weighing 4 kg consumes 5% of its body weight in food per day (its food intake rate). This leads to a PEC_oral rodent_ (Tier 1) of 25 mg/kg bw and PEC_oral, predator_ (Tier 2) of 1.25 mg/kg bw. On the effects side, the lowest reported acute toxicity of brodifacoum in cats was LD_50_ = 0.25 mg/kg [49,50]. For cats in our study, the RQ is calculated to be 1506. The calculations and applied assessment factors are provided in detail in the Supplementary Materials (Tables S8–S11).

## 4. Discussion

### 4.1. Prevalence of Rodenticides in Domestic Cats

This study shows for the first time the occurrence and concentration of rodenticides in domestic cats systematically at the country level, and which environmental and individual factors play a role. The AR residues of brodifacoum and bromadiolone, which are conspicuous in wildlife [8], are also conspicuous in cats. Based on the estimation of the cat population in Slovenia [29], around 290,000 cats carry residues of at least one rodenticidal active substance in their bodies.

The prevalence of total AR exposure in cats in the present study (*n* = 99) of 64.6%, based on the monitoring of liver concentrations, is significantly higher than the prevalence of 4.5% based on the monitoring of faecal concentrations found in a previous study [20]. This may be explained by the fact that the retention of ARs in faeces is shorter than in liver tissue and indicates acute rather than long-term (accumulative) exposure [16,52]. The results of our study are comparable to those of Koivisto et al. [22], but López-Perea et al. [23] recorded a lower prevalence and a higher mean value of pooled ARs. However, the small sample size in these multi-species studies makes such comparisons difficult.

Contrary to our hypotheses, there were no single-factor effects, but it was mainly the effects of human population density and rural region type that increased the residue occurrence and concentration of ARs. In densely populated rural areas, the presence of food in rubbish dumps, cultivated gardens, grain stores and livestock holdings (i.e., fodder) provides high food availability for commensal rodents. In addition, these man-made structures offer ample shelter for these animals. This may have necessitated the use of AR rodenticides to protect hygiene and animal health, especially in rural areas with a high human population density, which is reflected in the fact that brodifacoum concentrations are higher in rural landscapes than in intermediate ones. This differs somewhat from the situation in wild carnivores, where livestock density [36] and urban region type [23,36,38] play a role in this regard, but the results of this study confirm that human population density [39] can be a driver of AR residues in carnivores.

Somewhat surprising is the finding of a higher brodifacoum concentration in areas with a lower density of farms than in areas with higher farm density. At lower farm density, farms tend to be larger and more technologically advanced. Due to the closed systems and probably better rodent-proofing, rodents may find it more difficult to get inside the farms in such locations, especially on cattle farms. In larger chicken and pig farms, it is probably easier for rodents to access food, which could explain the exceptionally high maximum concentrations of brodifacoum in the Pomurska, Koroška and Goriška regions (Figure 3). Another reason for the higher number of compounds detected and the higher concentration of brodifacoum in areas with a lower density of farms could be that larger farms, as large economic enterprises, have to follow stricter regulations for rodent management (mandatory measures/certification in the food industry). This can lead to early action in the event of a new rodent infestation and more professional use of rodenticides, which ultimately leads to less use and thus less residues of ARs. Finally, rodents are more likely to occur sporadically on lower density farms than on higher density farms, where rodents are more evenly distributed and available to cats over a larger area.

Currently, five ARs and α-chloralose are authorised as biocides in Slovenia [53,54,55]. Interestingly, the detected prevalence of rodenticide residues in cat liver (Table S6) does not correlate with the number of authorised products on the Slovenian market (Table S12), indicating that the use of particular products with particular compounds is probably more important than the number of authorised products. In addition, the half-lives could also play a role.

The highest prevalence of brodifacoum residues of 53.5% stands out, which is also associated with a strong increase in its use [37], although it ranks second in terms of the number of authorised products in Slovenia (*n* = 53) (Table S12). Bromadiolone ranks second in prevalence (25.3%) and first in the number of authorised products (*n* = 70), while third in prevalence is the FGAR coumatetralyl (21.2%), which has only been authorised for 2 out of 163 products (1.2%) (Table S12). In Finland, the high prevalence of coumatetralyl is probably due to the 7.5- to 81-fold higher content in products compared to SGARs [22]. However, the coumatetralyl residue concentrations in cat livers in our study are low compared to those found for SGARs, with mean and maximum values of 1.52 and 4.1 ng/g, respectively (Table S6), which is likely due to the much shorter half-life (1.8–4 days) compared to SGARs (118–350 days) [22]. A high prevalence of coumatetralyl of 30% was also observed in feline faeces [20] and it was 48% in livers [22]. We also found that flocoumafen was not found in the cat liver samples at all, although its use has been confirmed among professional users [37]. Difethialone, found in cats with a prevalence of 2%, was registered in two biocidal preparations in Slovenia in the period 2021–2022 when the cats were sampled for this study, while it was no longer registered in 2024 (Table S12). Warfarin was detected in 1% of the samples, although it was not authorised at all in Slovenia at the time of sampling, suggesting the use of old stocks or possible illegal use after import from abroad.

The lack of individual effects was unexpected and is in contrast to the results found for cats delivered to an emergency service, where AR exposure is more likely in young cats and in cats living indoors [15]. The conflicting results may be due to the rather random origin of the cats used in this study, all of which spent at least some of their time outdoors and were not necessarily cared for by an owner and quickly hospitalised when symptoms of AR poisoning appeared.

The results of Cerkvenik-Flajs et al. indicate that foxes and domestic cats use the same food pool [37]. Considering the higher mean pooled AR concentration in foxes compared to cats in the present study (601 vs. 111 ng/g), we conclude that foxes may be more intensively exposed to AR poisoning than cats, and are therefore more vulnerable to the adverse effects of ARs [56].

### 4.2. Toxicological and Environmental Aspects

Clinical cases show that cats can ingest lethal amounts of ARs [57]. The single oral LD_50_ dose (mg/kg body weight) in cats of brodifacoum, bromadiolone and difenacoum is 0.25–25, 25 and 100 mg/kg body weight, respectively [49,58,59]. Compared to dogs, cats have a 10 to 25 times higher tolerance to the effects of some ARs, such as difenacoum, difethialone and bromadiolone, but this is reduced to a factor of 1 to 13 in the case of brodifacoum [60]. This is of concern, as brodifacoum was by far the most prevalent AR in the present study, found in 53.5% of cats (Figure 2, Table S6). Sublethal exposure to ARs can affect important protective physiological pathways of organisms, evidenced not only by impaired coagulopathy in bobcats, but also by decreased defence against extracellular pathogens and allergens, immune activation leading to exhaustion, increased cell death, decreased epithelial integrity and increased vulnerability to parasites [14]. However, a targeted study of healthy domestic cats aimed at assessing the effects of environmentally relevant sub-toxic brodifacoum exposure showed no impairment of their immune system [61]. The authors of the study cited genetic differences and the presence of numerous external stressors in the environment of feral cats, including exposure to multiple rodenticides, as possible reasons for their significant immune system dysregulation [61].

ARs can also cause anticoagulation-independent adverse effects such as impaired bone calcification, neuropathologic effects in the brain and nephropathologic effects, such as acute kidney injury, especially in patients with chronic kidney disease (CKD) [62]. In fact, kidney diseases are the most common diseases in cats [63], and the incidence of CKD in cats far exceeds that of other mammals [64], with an estimated prevalence of 1.6 to 20% in all age groups [65,66]. These facts indicate an increased risk of potential adverse effects of ARs in cats, with the development of haemoglobinuria and haematuria serving as early biomarkers of AR poisoning [62].

No haemorrhages linked to possible intoxication with ARs were noticed during necropsy of the animals studied, and those found were traumatic in origin. Low hepatic concentrations of ARs generally do not cause haemorrhagic bleeding, despite their extremely long half-life in the organism. Macroscopic haemorrhages may also be absent in animals with elevated and sub-toxic hepatic residue levels of ARs (>200 ng/g) [67].

Although we only found one positive sample containing α-chloralose, this shows that it is necessary to pay attention to this substance in the future, as it is also dangerous for cats. The significantly higher sensitivity of cats to α-chloralose compared to dogs is mainly due to the differences in their metabolisms, as cats have a limited capacity for ß-glucuronidation with respect to glucuronic acid conjugates, so the residence time of α-chloralose with toxic effects is longer in cats [68]. Additionally, cats are also more susceptible to hypothermia due to their larger body surface area to weight ratio [25].

Due to the lack of robust toxicity data for cats, it is extremely difficult to conduct a robust environmental risk assessment of secondary poisoning. The reported acute toxicity data for brodifacoum in cats range from 0.25 mg/kg body weight [49] to 25 mg/kg body weight (Rammell et al. [69], citing an anonymous source). For the worst case of acute brodifacoum poisoning, we calculated an RQ of 1506 for our study. This indicates a high risk of secondary poisoning based on the concentration in the bait. At a higher LD_50_, e.g., 25 mg/kg body weight, the RQ would be lower (here 15.06).

Comparing the calculated PECs and the determined brodifacoum concentrations in the cat livers is difficult, as the PEC is based on the body weight of the animal and not on the individual organ (liver). It is known, for example, that the liver is the main organ accumulating ARs, including brodifacoum, in mammals [70]. This is supported by data reported by Vandenbroucke et al. [71], who found that brodifacoum had the highest elimination half-life in the liver of mice compared to seven other ARs. Pharmacokinetic data for brodifacoum in cats are not available, but the results of Kopanke et al. [61] are in line with the assumption that cats, like their prey animals, accumulate brodifacoum mainly in the liver. This lack of robust data on the distribution and excretion of brodifacoum in cats leads to a high amount of uncertainty in the assessment of its chronic toxicity, and data from a chronic feeding study over 28 or 90 days would be required to clarify this. Furthermore, it is unknown whether the cats eat the whole prey (poisoned rodents) or only parts of it (liver or muscle).

## 5. Conclusions

This is the first study to systematically address and draw attention to the problem of asymptomatic poisonings of domestic cats with rodenticides from an environmental point of view. The finding that residues of at least one and up to four of the investigated substances were found in 65% of the analysed livers, with a maximum pooled concentration far exceeding the toxicological threshold for mammals, indicates a serious ecotoxicological problem not only in non-target wild animals but also in companion animals that are in contact with the environment. Urbanisation, rural region type and low density of farms were the main factors for environmental contamination with ARs when considering the number of substances found and their residue concentrations in the livers of cats.

Similar to in wildlife, residue levels of rodenticides in cats should be carefully monitored in the future. It is in the common interest to protect European ecosystems, including both wild and domestic animals, and this can only be done if the risks are known from monitoring data that reflect the diversity of European conditions, as wildlife does not respect national borders. To fully assess the threat to non-target animal systems and the environment, the amount of rodenticide products used should be monitored, particularly as many products are authorised for non-professional use (Table S12). Widespread use by non-professionals can trigger additional risk levels [22,37,56]. The risk assessment of chronic toxicity of rodenticides in non-target species also revealed a lack of necessary data on the distribution, excretion and feeding patterns of animals. The detailed information presented here on individual and landscape exposure patterns of non-target species in relation to AR application patterns could be used to derive and test risk mitigation environmental measures in the future [72].

## Figures and Tables

**Table 1:** Effects of landscape variables on log-brodifacoum concentrations. Confidence intervals and effect estimates are derived from bootstrapped Ordinary Least Squares (OLS) regression models (b = 10,000) to ensure robust estimation. CI—95% confidence interval; ref—reference.

	Estimate	Bootstrap CI	*p*-Value
Population density (ref: low)			
medium	0.54	[−1.04, 2.04]	0.48
high	1.13	[−0.55, 2.73]	0.16
Farm density (ref: low)			
medium	−0.62	[−2.11, 0.82]	0.37
high	−1.73	[−3.18, −0.12]	0.03
Livestock density (ref: low)			
medium	0.58	[−0.75, 1.79]	0.35
high	0.43	[−0.83, 1.73]	0.47
Region type (ref: intermediate)			
rural	1.42	[−0.18, 2.92]	0.07

**Table 2:** Effects of individual-level variables on log-brodifacoum concentrations. Confidence intervals and effect estimates are derived from bootstrapped Ordinary Least Squares (OLS) regression models (b = 10,000) to ensure robust estimation. CI—95% confidence interval; ref—reference.

	Estimate	Bootstrap CI	*p*-Value
Age (ref: 1–5 years)			
6–10 years	−0.58	[−1.91, 0.7]	0.38
11–15 years	−0.04	[−1.57, 1.7]	0.95
>15 years	−0.06	[−2.06, 1.25]	0.94
Sex (ref: female)			
male	−0.16	[−1.31, 1]	0.79
Activity (ref: inside/outside)			
outside	0.48	[−0.64, 1.61]	0.39

**Table 3:** Effects of individual-level variables on log-bromadiolone concentrations. Confidence intervals and effect estimates are derived from bootstrapped Ordinary Least Squares (OLS) regression models (b = 10,000) to ensure robust estimation. CI—95% confidence interval; ref—reference.

	Estimate	Bootstrap CI	*p*-Value
Age (ref: 1–5 years)			
6–10 years	−1	[−2.35, 0.49]	0.16
11–15 years	0.21	[−1.82, 1.69]	0.82
>15 years	−0.74	[−2.12, 1.14]	0.38
Sex (ref: female)			
Male	−0.45	[−1.68, 0.90]	0.49

## Data Availability

The data presented in this study are available on request from the corresponding author.

## Supplementary Materials

The following supporting information can be downloaded at https://www.mdpi.com/article/10.3390/toxics13080663/s1. Table S1: The data of the domestic cats (*Felis catus*), location data and the concentrations of ARs in the livers of the cats. Chemical residue analysis: Table S2: LC-MS/MS—MRM of the analytes, surrogates (Surr) and internal standards (IS) with precursor (Q1)–product ion (Q3) transitions; Table S3: Confirmation by Enhanced Product Ion spectra; Table S4: Reporting limit (RL) and recovery (Rec) ± relative standard deviation (RSD) of ARs in wild boar liver (*n* = 10); Table S5: Recovery (Rec) of surrogates over all domestic cat liver samples (*n* = 99) + relative standard deviation (RSD) with 100 ng/g wet weight. Results: Table S6: Descriptive statistics of the presence of anticoagulant rodenticides (ARs) and α-chloralose in the liver of domestic cats in Slovenia, collected in 2021 and 2022; Table S7: Presence of anticoagulant rodenticides (ARs) found in domestic cat livers in Slovenia in 2021 to 2022 according to region type. Environmental Risk Assessment of Secondary Poisoning by Brodifacoum: Table S8: Tier 1: Input parameters for the calculation of the concentration in prey (rodent) according to the ESD [48]; Table S9: Tier 2: Food intake for a cat according to the ESD [48]; Table S10: Input parameters for the calculation of the concentration in the predator according to the ESD [48]; Table S11: Input parameters for the calculation of the no adverse effect threshold (NAET). Authorised rodenticide products: Table S12: Anticoagulant rodenticide and α-chloralose products authorised in the Registry of Biocidal Products on the Market of Slovenia [55].

## Abbreviations

The following abbreviations are used in this manuscript:

AR	Anticoagulant rodenticide
EU	European Union
VKORC	Enzyme vitamin K epoxide reductase complex
FGAR	First-generation anticoagulant rodenticide
SGAR	Second-generation anticoagulant rodenticide
CNS	Central nervous system
LD	Lethal dose
LC-ESI-MS/MS	Liquid chromatography–electrospray tandem mass spectrometry
ERA	Environmental risk assessment
PEC	Predicted environmental concentration
PNEC	Predicted no-effect concentration
RQ	Risk quotient
ESD	Emission Scenario Document
FIP	Feline infectious peritonitis
FIV	Feline immunodeficiency virus
SD	Standard deviation
RR	Rate ratio
OR	Odds ratio
CI	Confidence interval
OLS	Ordinary Least Squares
CKD	Chronic kidney disease
