# Supplementary material for: Exposure of Domestic Cats (Felis catus) to Rodenticidal Compounds
Source: Toxics. 2025 Aug 7;13(8):663. doi: 10.3390/toxics13080663 (PMC12390110; doi:10.3390/toxics13080663)
Supplement: Supplementary file 1 [file toxics-13-00663-s001.zip › toxics-3737090-supplementary/250802 toxics-3737090-supplementary-proofed.docx]

**Chemical residue analysis**

**Table S2.** LC-MS/MS – MRM of the analytes, surrogates (Surr) and internal standards (IS) with precursor (Q1) – product ion (Q3) transitions.

| Analyte | Q1 Mass (m/z) | Q3 Mass (m/z) |
| --- | --- | --- |
| Chlorophacinone-d_4_ (IS) | 377 | 201 |
| Warfarin-d_5_ (IS) | 312 | 161 |
| Acenocoumarol (Surr) | 352* | 265* |
| Diphacinone-d_4_ (Surr) | 343* | 167* |
| Brodifacoum | 521 | 78.8 |
|  | 521 | 135 |
|  | 521* | 93* |
| Bromadiolone | 527* | 250* |
|  | 525 | 181 |
|  | 525* | 79* |
| Chlorophacinone | 373* | 201* |
| Coumatetralyl | 291* | 141* |
|  | 291 | 247 |
| Difenacoum | 443* | 135* |
|  | 443 | 93 |
| Difethialone | 539* | 80.8* |
|  | 539 | 151 |
| Flocoumafen | 541* | 382* |
|  | 541 | 161 |
| Warfarin | 307* | 161* |
|  | 307 | 250 |
| α-Chloralose | 307* | 161* |
|  | 307 | 100.8 |

*Quantifying transition

**Table S3**. Confirmation by Enhanced Product Ion spectra.

| EPI (enhanced product ion spectra) | | | | | |
| --- | --- | --- | --- | --- | --- |
| Mass range |  | DP | | EP | CE |
| (m/z) |  | (V) | | | |
| 50 – 450 m/z |  | -50 | -10 | | -30 ( ±15) |

The threshold value for the acceptance of an AR was an agreement of more than 80% between the enhanced product spectra of the sample and the corresponding standards.

In each batch of the analysis of the 99 liver samples from domestic cats, recovery samples were included for quality control. Blank wild boar liver was used as the matrix and fortified with 100 ng/g of each analyte (Table S4).

**Table S4**. Reporting limit (RL) and recovery (Rec) ± relative standard deviation (RSD) of ARs in wild boar liver (n = 10); in control samples (n = 2), all not detected; n. d. (not detected) = < Reporting Limit (RL).

| Analyte | RL | 100 ng/g | |
| --- | --- | --- | --- |
|  | (ng/g) | Rec  (%) | RSD  (%) |
| Brodifacoum | 2.0 | 68 | 21 |
| Bromadiolone | 2.0 | 86 | 13 |
| Chlorophacinone | 10.0 | 95 | 13 |
| Coumatetralyl | 0.2 | 92 | 10 |
| Difenacoum | 2.0 | 87 | 18 |
| Difethialone | 2.0 | 63 | 27 |
| Flocoumafen | 2.0 | 79 | 24 |
| Warfarin | 0.2 | 103 | 6 |
| α-Chloralose | 10.0 | 102 | 8 |
| Acenocoumarol (Surr) |  | 110 | 10 |
| Diphacinone-d_4_ (Surr) |  | 107 | 9 |

All domestic cat liver samples were spiked with the surrogate mixture for ongoing validation of analytical performance (Table S5).

**Table S5**. Recovery (Rec) of surrogates over all domestic cat liver samples (n = 99) + relative standard deviation (RSD) with 100 ng/g wet weight.

| Surrogates |  | 100 ng/g | |
| --- | --- | --- | --- |
|  | n  99 | Rec  (%) | RSD (%) |
| Acenocoumarol Brodifacoum |  | 97 | 14 |
| Diphacinone-d_4_ |  | 134 | 36 |

The different results for acenocoumarol and diphacinone-d_4_ in the quality analytical (QA) samples compared to the analyses of field samples may be due to their different origins.

**Results**

**Table S6**. Descriptive statistics of the presence of anticoagulant rodenticides (ARs) and α-chloralose in the liver of domestic cats in Slovenia, collected in 2021 and 2022. n—number of samples with residues, Min—minimum concentration, Max—maximum concentration, SD—standard deviation, FGARs—first-generation anticoagulant rodenticides, SGARs—second-generation anticoagulant rodenticides. ARs chlorophacinone and flocoumafen are excluded from the table as they were not found.

| Sample size = 99 | n | % | Min (ng/g) | Max (ng/g) | | Mean (ng/g) | Median (ng/g) | SD |
| --- | --- | --- | --- | --- | --- | --- | --- | --- |
| α-chloralose | 1 | 1.0 | 561.71 | | 561.71 | 561.71 | 561.71 |  |
| Brodifacoum | 53 | 53.5 | 2.65 | | 1,802.82 | 116.69 | 22.13 | 295.65 |
| Bromadiolone | 25 | 25.3 | 2.13 | | 183.70 | 30.06 | 6.05 | 48.86 |
| Coumatetralyl | 21 | 21.2 | 0.30 | | 4.08 | 1.52 | 1.48 | 0.88 |
| Difenacoum | 8 | 8.1 | 2.54 | | 41.56 | 15.07 | 12.54 | 12.76 |
| Difethialone | 2 | 2.0 | 2.66 | | 6.23 | 4.44 | 4.44 | 2.52 |
| Warfarin | 1 | 1.0 | 14.15 | | 14.15 | 14.15 | 14.15 |  |
| All ARs | 64 | 64.6 | 0.80 | | 1,819.74 | 110.75 | 24.27 | 274.09 |
| FGARs (2 ARs) | 22 | 22.2 | 0.30 | | 14.15 | 2.09 | 1.55 | 2.83 |
| SGARs (4 ARs) | 55 | 55.6 | 2.65 | | 1,819.74 | 128.47 | 44.63 | 292.11 |

All ARs—sum of all ARs for those cats that contained at least one AR.

FGARs—sum of all first-generation ARs (2 FGAR) for those cats that contained at least one FGAR.

SGARs—sum of all second-generation ARs (4 SGAR) for those cats that contained at least one SGAR.

**Table S7**. Presence of anticoagulant rodenticides (ARs) and α-chloralose found in domestic cat livers in Slovenia in 2021 to 2022 according to region type.

| Intermediate region type | | | |  | Predominantly rural region type | | | |
| --- | --- | --- | --- | --- | --- | --- | --- | --- |
| Regions (with data): Gorenjska, Obalno-kraška, Osrednjeslovenska | | | |  | Regions (with data): Goriška, Jugovzhodna Slovenija, Koroška, Podravska, Pomurska, Savinjska, Spodnjeposavska | | | |
| 22 of 43 samples AR-positive (51.2%)  (inside Gorenjska region 85.7%, inside others below 50% positive) | | | |  | 42 of 56 samples AR positive (75.0%)  (inside Savinjska region 50% positive, inside all other regions above 70% positive,  1 outdoor male from Goriška region with α-chloralose) | | | |
|  | positive | Maximum concentration (ng/g) | Total concentration (ng/g) |  |  | positive | Maximum concentration (ng/g) | Total concentration (ng/g) |
| Brodifacoum | 19 | 271.65 | 1184.17 |  | Brodifacoum | 34 | 1802.82 | 5000.50 |
| Bromadiolone | 8 | 46.17 | 153.55 |  | Bromadiolone | 17 | 183.70 | 597.92 |
| Coumatetrayl | 8 | 2.45 | 10.80 |  | Coumatetralyl | 13 | 4.08 | 21.05 |
| Difenacoum | 2 | 6.59 | 9.13 |  | Difenacoum | 6 | 41.56 | 111.46 |
| Difethialone | 1 | 6.23 | 6.23 |  | Difethialone | 1 | 2.66 | 2.66 |
| Warfarin | 0 | 0 | 0 |  | Warfarin | 1 | 14.15 | 14.15 |
| Difethialone was found in a young female cat in the Obalno-kraška region with  traces of three ARs (besides difethialone, also brodifacoum and coumatetralyl). | | | |  | All max concentrations are higher than for intermediate region type except difethialone. Difethialone was found in an old cat in Podravska region that had traces of four ARs. | | | |

**Environmental Risk Assessment of Secondary Poisoning by Brodifacoum**

*Exposure estimation according to ESD document PT 14 [48], chapter 5.5.2*

**Table S8**. Tier 1: Input parameters for the calculation of the concentration in prey (rodent) according to the ESD (chapter 5.5.2.1, Table 36 [48].

| **Parameters** | **Nomenclature** | **Value** | **Unit** | **Origin** | | **Remarks** |
| --- | --- | --- | --- | --- | --- | --- |
| Input | | | | | |  |
| Food intake rate/body weight rodent | FIR/BW_rodent_ | 0.1 | [g food.g^-1^ bw.d^-1^] | | D | Default |
| Concentration of the active substance in the fresh diet (bait) | C | 50 | [mg.kg^-1^] | | S | BPC opinion on brodifacoum [50] |
| Avoidance factor | AV | 1 | [-] | | D | Default |
| Fraction of diet obtained in treated area | PT | 1 | [-] | | D | Default |
| Composition of diet obtained in treated area | PD | 1 | [-] | | D | Default |
| ADME factor | ADME | 0 | [-] | | D | Default |
| Number of days the rodent is consuming rodenticide | N | 4 | [d] | | D | Worst case: 5 consecutive days |
| Fraction of poisoned rodents in predators’ diet | F_rodent_ | 1 | [-] | | D | Acute poisoning: 100% of the daily diet.  Chronic: 0.5 |
| Output | | | | | |  |
| Concentration in food (rodent) after one day | C_food, rodent_ | 5 | [mg.kg^-1^ food.d^-1^] | | O | Equation 1 |
| Predicted environmental concentration of an active substance in the food (= rodent) of a predator/scavenger | PEC_oral, rodent_ | 25 | [mg.kg^-^1 food] | | O | Equation 2 |

Origin: D—default; O—output; S—data set (substance specific value)


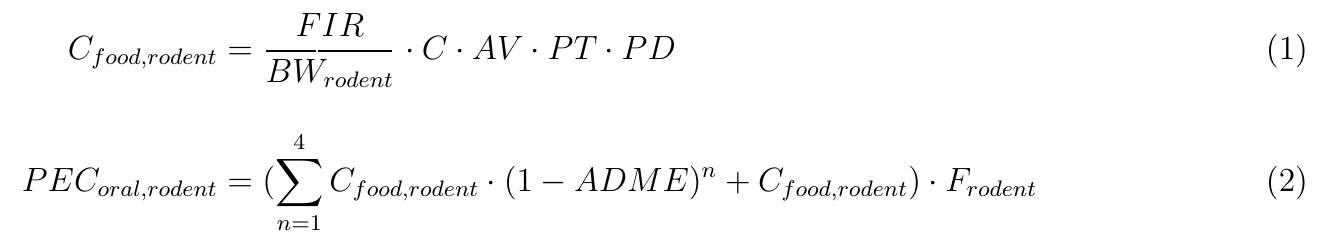


**Table S9**. Tier 2: Food intake for a cat according to the ESD (chapter 5.5.2.2 [48]), excluding Table 37, p. 90.

| **Generic focal non-target species** | **Prey** | **Body weight (g)** | **Daily mean fresh food intake (g)** | **Food intake rate / body weight (g.g^-1^ bw per day)** |
| --- | --- | --- | --- | --- |
| Domestic cat (*Felis silvestris catus*) | rodent | 4,000 | 200 | 0.05 |

Note: Values calculated based on a cat’s daily maintenance energy requirement of 60 kcal/kg bw. A mouse contains approximately 30 kcal. Assuming a mouse bw of 25 g, this results in a 200 g fresh mouse diet.

**Table S10**. Input parameters for the calculation of the concentration in the predator according to the ESD, Table 38 [48].

| **Parameters** | **Nomenclature** | **Value** | **Unit** | **Origin** | **Remark** |
| --- | --- | --- | --- | --- | --- |
| Input | | | | |  |
| Predicted environmental concentration of an active substance in the food (= rodent) of a predator/scavenger | PEC_oral, rodent_ | 25 | [mg.kg^-1^ bw] | O | Result from Table 36 |
| Food intake rate / body weight | FIR/BW | 0.05 | [g food.g^-1^ bw.d^-1^] or  kg/kg bw per d | P | See Table 37 |
| Output | | | | |  |
| Predicted environmental concentration of an active substance in a rodent predator per day | PEC_oral, rodent predator_ | 1.25 | [mg.kg^-1^ bw.d^-1^] | O |  |

Origin: O—output; P—picklist


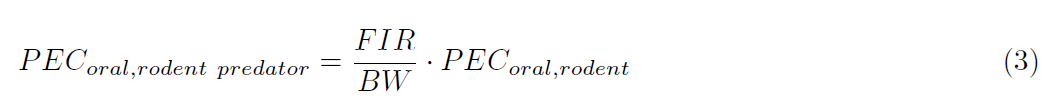


Following the procedure as detailed in the ESD [48], it is assumed that a predator prey (i.e., a target animal mouse or rat) forages on bait only for five consecutive days and is caught by the predator (in the present case, a cat) immediately after their last meal on day five. It is further assumed that rodents consume 10% of their body weight in food per day, and that a cat weighing 4 kg consumes 5% of its body weight food per day (its food intake rate). This leads to a PEC_oral rodent_ (Tier 1) of 25 mg/kg bw and PEC_oral, predator_ (Tier 2) of 1.25 mg/kg bw.

*Data on acute effects in cats*

**Table S11**. Input parameters for the calculation of the no adverse effect threshold (NAET).

| **Parameters** | **Nomenclature** | **Value** | **Unit** | **Origin** | **Remark** |
| --- | --- | --- | --- | --- | --- |
| Input | | | | |  |
| Acute oral lethal dose | LD_50_ | 0.25 | [mg.kg^-1^ bw] | P | Eason and Wickstrom [49] |
| Assessment factor | AF | 300 | [-] | P | ESD [48] |
| Output | | | | |  |
| No adverse effect threshold | NAET | 8.3 10^-4^ | [mg.kg^-1^ bw] | O |  |

Origin: O—output, P—picklist


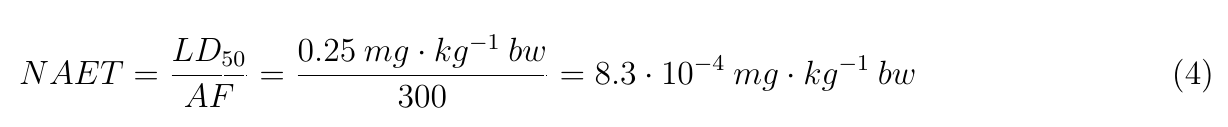


On the effects side, the lowest reported acute toxicity of brodifacoum in cats is LD_50_ = 0.25 mg/kg [49]. To derive a no adverse effects threshold (NAET), an assessment factor (AF) of 300 is required by the ESD [48]. This is to cover uncertainties due to extrapolation from lab to field and extrapolation from acute LD_50_ to the no observable effects concentration (NOEC).

*Risk characterisation*

To assess the risk, PEC and NAET values are compared, and their quotient (risk quotient, RQ) is calculated. An RQ equal to or greater than 1 indicates an unacceptable risk to the environment, with a higher RQ indicating a greater risk.


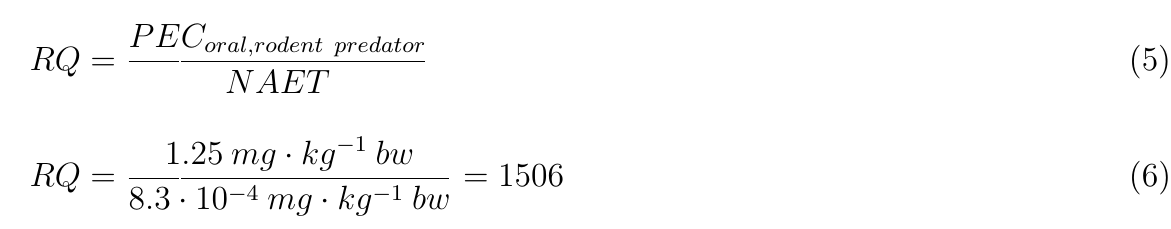


**Authorised rodenticide products**

**Table S12**. Anticoagulant rodenticide and α-chloralose products authorised in the Registry of Biocidal Products on the Market of Slovenia [55].

| Active substance | Number of products on the market | Category of use and concentration of active substance(s) in bait | | |
| --- | --- | --- | --- | --- |
|  |  | Only professional  use | Both professional and  general (non-professional) use | Only general use |
| α-Chloralose | 16 | 1 | 15 | - |
|  |  | 4% | 4% |  |
| Brodifacoum | 53 | 11 | 42 | - |
|  |  | 0.005% | 0.0025% (22) |  |
|  |  |  | 0.0029% (19) |  |
|  |  |  | 0.005% (1) |  |
| Bromadiolone | 70 | 49 | 17 | 4 |
|  |  | 0.005% (42) | 0.0025% (2) | 0.0029% |
|  |  | 0.032% (7) | 0.0027% (9) |  |
|  |  |  | 0.0029% (4) |  |
|  |  |  | 0.005% (2) |  |
| Coumatetralyl | 2 | 1 | - | 1 |
|  |  | 0.406% |  | 0.0026% |
| Difenacoum | 14 | 12 | 2 | - |
|  |  | 0.005% | 0.0025% |  |
| Difenacoum and bromadiolone | 1 | 1 | - | - |
|  |  | 0.0025% |  |  |
| Flocoumafen | 7 | - | 7 | - |
|  |  |  | 0.0025% |  |
| Sum | 163 | 75 | 83 | 5 |

**References**

1. European Chemicals Agency (ECHA). *Revised Emission Scenario Document for Product Type 14. Rodenticides.* ECHA-18-H-23-EN. Helsinki, Finland: ECHA, 2018. ISBN 978-92-9020-837-2. https://doi.org/10.2823/660595.
2. Eason, C.T., Wickstrom, M. *Vertebrate Pesticide Toxicology Manual (poisons): Information on Poisons Used in New Zealand as Vertebrate Pesticides*. Wellington, New Zealand: Department of Conservation, P.O. Box 10-420, 2001. Available online: <https://www.doc.govt.nz/documents/science-and-technical/docts23.pdf> (accessed on 14 March 2025).
3. European Chemicals Agency (ECHA). *Opinion on the application for renewal of the approval of the active substance: Brodifacoum Product type: 14* ECHA/BPC/113/2016, 2016. Available online: <https://echa.europa.eu/documents/10162/b85dfd6e-177b-43df-809c-180bc025b612>. (accessed on 14 March 2025).
4. [dataset] Republic of Slovenia. Register of biocidal products on the market of the Republic of Slovenia, Ministry of Health Office of the Republic of Slovenia for Chemicals, 2024. <https://podatki.gov.si/dataset/register-biocidnih-proizvodov-na-trgu-rs>. (accessed on 8 April 2024).
